# Case report: Bullous pemphigoid arising in a patient with scleroderma and multiple sclerosis

**DOI:** 10.3389/fmed.2022.1055045

**Published:** 2022-12-08

**Authors:** Francesco Moro, Feliciana Mariotti, Anna Pira, Naomi De Luca, Biagio Didona, Gianluca Pagnanelli, Giovanni Di Zenzo

**Affiliations:** ^1^Dermatology Unit, Istituto Dermopatico dell’Immacolata (IDI)-IRCCS, Rome, Italy; ^2^Molecular and Cell Biology Laboratory, IDI-IRCCS, Rome, Italy

**Keywords:** bullous pemphigoid, scleroderma, multiple sclerosis, BP180, BP230

## Abstract

**Background:**

Bullous pemphigoid (BP) is the most common autoimmune-blistering disease, clinically characterized by erythematous urticarial plaques, blisters, and intense pruritus, induced by autoantibodies against two proteins of the dermo-epidermal junction, BP180 and BP230. A large number of autoimmune diseases are reported in the literature as BP comorbidities, such as multiple sclerosis, but only a few cases are in association with scleroderma and none in association with both.

**Case presentation:**

We present the case of a 68-year-old woman affected by multiple sclerosis and scleroderma who developed severe bullous pemphigoid with a bullous pemphigoid disease area index of 60 and high titers of anti-BP180 and anti-BP230 autoantibodies by enzyme-linked immunosorbent assays. After 2 months of therapy with both intravenous and oral corticosteroids, the active lesions of bullous pemphigoid were remitted with no relapse.

**Conclusion:**

Autoimmune diseases affecting the skin or organs where BP180 and BP230 are present could trigger an immune response to these antigens through an epitope-spreading phenomenon and, over the years, induce bullous pemphigoid onset.

## Introduction

Bullous pemphigoid (BP) is the most common autoimmune-blistering disease, induced by autoantibodies against two proteins of the dermo-epidermal junction, BP180 and BP230. The binding of the autoantibodies results in an inflammatory response with complement activation, degranulation of mast cells, accumulation of neutrophils and eosinophils, and release of proteolytic enzymes that cleave BP180 and lead to blister formation. Clinically, BP is characterized by intense pruritus, erythematous urticarial plaques, and blisters. The diagnostic gold standard is the confirmation with direct immunofluorescence (DIF) of linear deposition of IgG and/or C3 along the dermo-epidermal junction. Other useful assays are indirect immunofluorescence to reveal IgG staining of the dermo-epidermal junction, enzyme-linked immunosorbent assays (ELISA) to detect autoantibodies against BP180 and BP230, and the histological examination of lesional skin biopsy to evaluate dermo-epidermal detachment and inflammatory infiltrate. A large number of autoimmune diseases are reported in the literature as BP comorbidities: psoriasis, rheumatoid arthritis, lupus erythematosus, lichen planus, membranous nephropathy, pernicious anemia, primary biliary cirrhosis, thyroiditis, polymyositis, and multiple sclerosis (MS) (1, 2). On the other hand, only a few cases of BP associated with connective tissue disorders, such as systemic sclerosis, also termed scleroderma (SCL) (two cases) or morphea (three cases), are reported, and no case of BP has been described in association with both MS and SCL, so far (3–7).

## Case description

**FIGURE 1:**
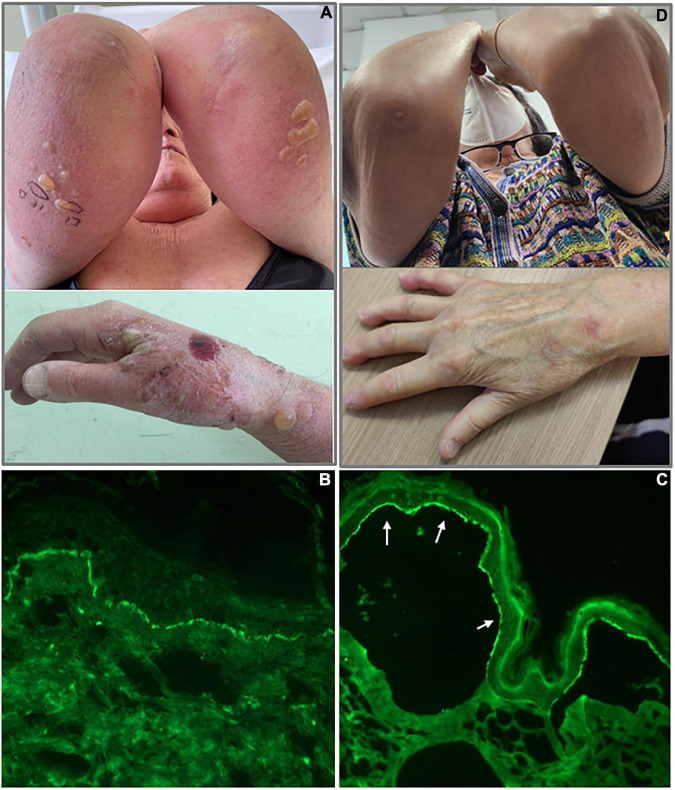
Clinical and immunological findings of bullous pemphigoid patient. **(A)** Tense bullae in the upper limbs and the right hand; **(B)** linear deposition of IgG along the dermo-epidermal junction by direct immunofluorescence; **(C)** IgG staining of the epidermal side (white arrows) of the artificial blister by indirect immunofluorescence on human salt-split skin; and **(D)** patient with BP in complete remission on minimal therapy.

In February 2022, a 68-year-old woman came to our clinic for evaluation of bullous and urticarial lesions. On physical examination, the patient showed excoriations and tense bullae on an inflammatory basis in the upper limbs, lower limbs, trunk, and abdomen and a lesion in the nasal mucosa, with a bullous pemphigoid disease area index (BPDAI) score of 60 (Figure 1A). She also showed sclerodactyly with Raynaud’s phenomenon, tightening facial skin with telangiectasias, thin lips, and perioral wrinkles. At physical examination, no other remarkable sign was detected. She had no family history of autoimmune-blistering disease or different skin inflammatory diseases. The findings of routine laboratory tests, chest radiography, and abdominal computed tomography scan were within normal limits.

**FIGURE 2:**
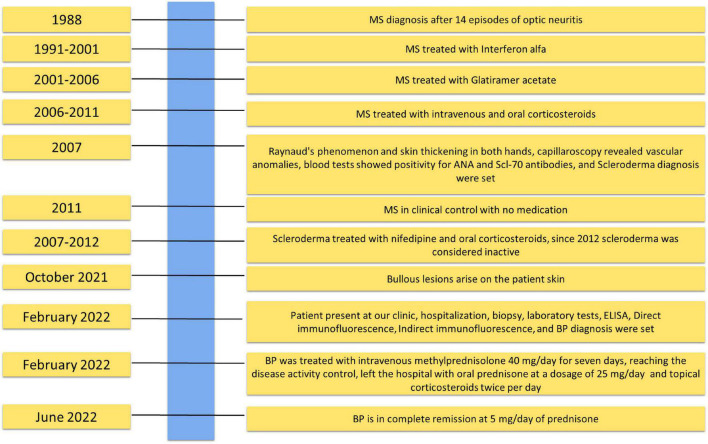
Timeline with relevant data about the onset, diagnosis, and therapy of bullous pemphigoid patient.

The patient reports that bullous lesions started in October 2021 in a milder form, and she initially went to a private practice dermatologist who set therapy with betamethasone 5 mg/day (Figure 2). The lesions remitted during the treatment and then relapsed in a more severe form with an unbearable itching in the trunk and limbs once the intake of corticosteroids was stopped.

Medical history has revealed that the patient is also affected by MS, diagnosed in 1988 following 14 episodes of optic neuritis: magnetic resonance imaging and lumbar puncture showing oligoclonal bands confirmed the diagnosis (Figure 2). She was treated from 1991 to 2001 with interferon alfa and subsequently from 2001 to 2006 with Glatiramer acetate 20 mg/die thrice per week. Thereafter, she underwent only oral or intravenous corticosteroids. Since 2011, the MS has been in clinical control, and the patient does not use any medication.

In addition, in 2007, the patient was evaluated for Raynaud’s phenomenon and skin thickening in both hands. Capillaroscopy revealed vascular anomalies, such as mega capillaries and avascular areas (Figure 2). Subsequent blood tests showed positivity for ANA and Scl-70 antibodies. The diagnosis of SCL was set, though the computed tomography scan was negative for other organ damage, and therapy with nifedipine and oral corticosteroids 10 mg/die was set up. After 5 years, the treatment was suspended because the disease was considered not active.

We decided to hospitalize the patient, and a blood draw and skin biopsy were performed. Histological examination showed a subepidermal detachment, with inflammatory infiltrate of neutrophils and eosinophils. DIF was positive with linear deposition of IgG and C3 along the dermo-epidermal junction (Figure 1B). We performed indirect immunofluorescence on human salt-split skin and BP180 and BP230 ELISAs. Both the immunological assays resulted in positive with IgG staining of the epidermal side of the artificial blister by IIF (Figure 1C) and IgG titers of 36.4 and 44.1 U/ml against BP180 and BP230, respectively (negative <9 U/ml for both BP180 and BP230 ELISA) (MBL, Nagoya, Japan).

The laboratory data, the biopsy outcome, and the suggestive clinic features allowed us to diagnose BP. Of note, at the time of the BP diagnosis, the patient was not receiving medication for MS and SCL or other diseases.

We decided on therapy with intravenous corticosteroids and methylprednisolone 40 mg/day for 7 days. After 7 days, the patient’s BPDAI dropped to 33 with no new lesions reaching disease activity control.

The patient left the hospital and continued to take oral prednisone at a dosage of 25 mg/day and to use topical therapy (Clobetasol propionate 0.05% cream) twice a day on lesions. No other steroid-sparing medication was used. After 2 months, she reached remission on minimal therapy of 10 mg/day of prednisone, and a BPDAI of 10 was only obtained with damage evaluation (Figure 1D). Two months later, she was in complete remission at 5 mg/day of prednisone with a BPDAI of 3. In the present case, the prompt response to therapy could be related to the absence of comorbidities and good general condition. The patient reported no problems returning to regular daily activity, with no physical or psychological sequelae since she reached remission on minimal therapy.

## Discussion

We presented a case of BP arising in a patient affected by two different systemic autoimmune diseases. MS is a demyelinating autoimmune disease that has been associated with the onset of BP in various studies (8, 9). A recent meta-analysis defined a relative risk of MS among patients with BP of 12.40 (2). The mechanism behind this association remains unknown. It has been found that the genes of BP target antigens are expressed in the central nervous system, so it has been assumed that repeated insults to the organ can induce an epitope-spreading phenomenon and trigger an autoimmune response in predisposed individuals. Several studies are needed to elucidate this mechanism better.

The SCL is a rare autoimmune connective tissue disorder presenting with cutaneous sclerosis and systemic involvement, while morphea is a localized scleroderma confined to the skin and/or underlying tissues. A few cases of BP associated with SCL or morphea have been described in the past. In most reported cases, the association appears to be occasional, often explained as a consequence of Koebner’s phenomenon or as an association/adverse effect following drug therapy. Therefore, given the small number of cases and other factors that could intervene, the association was considered causal (5–7). More recently, Cozzani et al. also reported a BP occurring in a patient with SCL. However, the patient had recently taken piperacillin and, according to the Naranjo algorithm, the diagnosis was consistent with drug-induced BP (4). In 2022, Maglie et al. reported a case of BP in a patient with a long history of morphea and lichen sclerosus. Interestingly, they report the BP lesions were localized to the same area previously affected by morphea and lichen sclerosus. The authors hypothesized that morphea and lichen sclerosus could be two predisposing factors for the development of BP due to a T-cell reactivity to BP180 NC16A and a Th2-type signaling activation (3).

In our patient, BP developed 34 years after the diagnosis of MS and 14 years after the diagnosis of SCL. In the absence of other events that may have acted as trigger factors, such as drug intake, trauma, radiation, recent infections, and UV exposure, we can hypothesize that the two pre-existing autoimmune diseases acted as predisposing factors. In general, the impairment of the immune system based on MS and SCL could provide the immunological background for the induction of BP. In addition, the autoimmune response could lead to chronic tissue damage inducing the activation and recruitment of lymphocytes specific for epitopes/antigens, which are distinct from and non-cross-reactive with the disease-inducing epitope/antigen. In this context, one of the target tissues for SCL is the skin, where BP antigens, BP180 and BP230, are structural components. In parallel, in MS, the target of an autoimmune response is the central nervous system, where BP180 and BP230 expressions have been demonstrated (10–12). In addition, anti-BP180 and anti-BP230 autoantibodies were detected in the cerebrospinal fluid of patients with BP and neurological diseases (13). Immunosenescence may have a role in the development of BP, and in our case, it may have been a crucial trigger factor (1). However, not all aged patients affected by MS and/or SCL develop BP, suggesting that other specific predisposing factors are needed. It could be hypothesized that the pre-existence of low titers of circulating autoantibodies arising after chronic exposure to antigens may have favored BP development in our aged patient.

The strength of our case is the unicity; as we know, this is the first reported case of MS, scleroderma, and BP arising in the same patient. As for the limitations, we do not have a blood sample before the BP onset, and we cannot state the presence of low-titer autoantibodies before the clinical presentation.

## Conclusion

This case could lead to thinking that autoimmune diseases affecting the skin or organs where genes coding for BP180 and BP230 are expressed could, over time, induce the exposure of antigens, stimulate a specific activation of the immune system, and consequently lead to the development of BP.

## Data availability statement

The raw data supporting the conclusions of this article will be made available by the authors, without undue reservation.

## Ethics statement

The studies involving human participants were reviewed and approved by the Ethical Committee of Istituto Dermopatico dell’Immacolata (IDI) IRCCS. The patients/participants provided their written informed consent to participate in this study. Written informed consent was obtained from the individual(s), and minor(s)’ legal guardian/next of kin, for the publication of any potentially identifiable images or data included in this article.

## Author contributions

GD, GP, and BD conceived and design the study. GD and FMo wrote the manuscript. FMa, AP, and ND collected the data and performed the analysis and experiments. All authors contributed to the article and approved the submitted version.
